# Biologics and biosimilars medication errors in Saudi Arabia: a nationwide retrospective observational real-world study

**DOI:** 10.3389/fmed.2026.1795867

**Published:** 2026-04-07

**Authors:** Nouf T. Alshammri, Wafa S. Alluwaymi, Ghadah A. Assiri, Thamir M. Alshammari

**Affiliations:** 1 Therapeutic Affairs Deputyship, Ministry of Health, Riyadh, Saudi Arabia; 2 Department of Clinical Pharmacy, College of Pharmacy, King Saud University, Riyadh, Saudi Arabia; 3 Department of Pharmacy Practice, College of Pharmacy, Jazan University, Jazan, Saudi Arabia; 4 Pharmacy Practice Research Unit, College of Pharmacy, Jazan University, Jazan, Saudi Arabia

**Keywords:** biologicals, biosimilars, healthcare professionals, medication error, pharmacovigilance, Saudi Arabia

## Abstract

**Background:**

In Saudi Arabia, the Ministry of Health reported 71, 332 errors from 2018 to 2019. Biologics and biosimilars are safe treatment options for various medical conditions; however, safety concerns remain. Immunogenicity and unique brand names can cause adverse reactions, and the ability to predict immune responses is limited. This study aims to determine the number and characteristics of medication errors associated with biological and biosimilar medications in Saudi Arabia from 2020 to 2023.

**Methods:**

A retrospective observational real-world study was conducted to analyze the number of biologic and biosimilar medication errors reported to the MOH, Saudi Arabia, from 2020 to 2023 using an electronic database as a data source. Categorical data were summarized as frequency and percentage. All analyses were performed using Microsoft Excel.

**Results:**

A total of 63,817 medication error reports related to biologics and biosimilars were identified. The majority occurred during the prescribing phase (76–79%) and were classified as non-harmful (Category B, ≈70%). Pharmacists were the leading reporters (87%), underlining their central role in patient safety. Adults (>18 years) represented the most affected demographic group (≈70%). Insulin and anticoagulants (enoxaparin and heparin) together accounted for the highest proportion of errors, highlighting these agents as key targets for safety interventions.

**Conclusion:**

This study offers a comprehensive national evaluation of medication errors associated with biologic and biosimilar products reported to the Saudi Ministry of Health between 2020 and 2023. High-alert medications, particularly insulin and anticoagulants, consistently ranked among the top error-related agents, highlighting the need for targeted interventions.

## 1 Introduction

It is generally acknowledged that medication errors are the most frequent and preventable cause of patient harm (1). The National Coordinating Council for Medication Error Reporting and Prevention (NCCMERP) defines a medication error as follows: “A medication error is any preventable event that may cause or lead to inappropriate medication use or patient harm while the medication is in the control of the health care professional, patient, or consumer. Such events may be related to professional practice, health care products, procedures, and systems, including prescribing, order communication, product labeling, packaging, and nomenclature, compounding, dispensing, distribution, administration, education, monitoring, and use” (2). Medication errors are a widespread global issue. On an annual basis, more than 100,000 reports of possible medication errors are submitted to the Food and Drug Administration (FDA) of the United States of America (3). Every year in England, 237 million medication errors occur (4). From March 2018 to June 2019, the Ministry of Health (MOH) of Saudi Arabia reported 71,332 medication errors. The MOH is responsible for more than half of all healthcare services in Saudi Arabia (5). Biologics and biosimilars are safe and effective treatment options for a wide range of medical conditions, including arthritis, kidney disease, cancer, and chronic skin and gastrointestinal disorders (such as psoriasis, irritable bowel syndrome, Crohn’s disease, and colitis) (6). The FDA defines a biological product as follows: “Biologics are generally large, complex molecules that are made from living sources such as bacteria, yeast, and animal cells” (6). Several significant safety concerns have been identified in the post-marketing phase of biologics use. In general, adverse events associated with these agents are attributable to an enhancement of the known pharmacologic actions, such as the risk of infections and malignancies, or to immunologic and infusion reactions, including the development of anti-drug antibodies due to the protein nature of these agents (7). Due to the high cost of this biological product and the recent expiration of the patents on a large number of them, pharmaceutical companies have been encouraged to investigate the possibility of producing biosimilars (8). The FDA defines a biosimilar product as follows: “A biosimilar is a biologic medication that is highly similar to and has no clinically meaningful differences from an existing FDA-approved biologic, called a reference product” (6). Biosimilars have the potential to increase competition among manufacturers, decrease costs, and increase patient access to medications (8). Furthermore, Biosimilars saved $7 billion in 2021 and $13 billion since 2015 (9). Therefore, biosimilars may usher in a new era of chronic illness in the coming decades (8). However, there are two major safety concerns regarding biosimilars. First, immunogenicity is the potential risk of adverse immune reactions to a drug. Biosimilars can directly induce drug antibodies because of their molecular size, which can seriously affect their safety and efficacy. Moreover, the ability to predict immune responses in patients is limited (10, 11). Second, the naming of biosimilar products is a challenge. The World Health Organization (WHO) recommends that biosimilars have unique brand names and lot numbers to ensure traceability. However, unique names may confuse prescribers, leading to medication errors and adverse events (11). Although biosimilars are biologic products by definition, they are approved through distinct regulatory pathways compared to originator biologics. International regulatory comparisons between the United States FDA and the European Medicines Agency (EMA) demonstrate differences in authorization frameworks, interchangeability policies, and post-marketing pharmacovigilance requirements that may influence prescribing and medication-use processes (12). In the United States (US), reference biologics are licensed under section 351(a) of the Public Health Service Act. In contrast, biosimilars are approved under the abbreviated 351(k) pathway, which requires demonstration of high similarity without clinically meaningful differences (3). Similarly, the SFDA regulates biosimilars under a dedicated regulatory framework that specifies quality and comparability requirements distinct from those applied to originator biologics (13). Therefore, while biosimilars are biologic medicines from a scientific perspective, they are treated as distinct regulatory and market entities within healthcare systems. This distinction supports their separate analytical characterization in the present study. Analyzing and learning from errors are among the most important steps in reducing risk and improving patient safety. Furthermore, once safety concerns are identified, the necessary changes are implemented, resulting in a much broader appreciation of the value of the systems approach to error prevention (1). To date, limited published data have examined medication errors specifically associated with biologic and biosimilar medications within unified national reporting systems, particularly in the Saudi context. The study aimed to determine the medication errors associated with biologic and biosimilar medications and the magnitude of those errors reported to the MOH of Saudi Arabia from 2020 to 2023. Also, to determine the stage and category of the error, identify the patients’ age and gender group, identify the group of healthcare professionals who reported the errors, and determine the possible cause and contributing factors for the medication error.

## 2 Materials and methods

### 2.1 Study design

A retrospective observational real-world study was conducted to analyze all biologic and biosimilar medication errors reported to the General Department of Pharmaceutical Care at the MOH, Saudi Arabia, from 2020 to 2023. This study followed the Strengthening the Reporting of Observational Studies in Epidemiology (STROBE) checklist and the Reporting of Studies Conducted Using Observational Routinely Collected Health Data (RECORD) statement (14, 15).

### 2.2 Study setting

Data on all biologic and biosimilar medication errors were reported to the General Department of Pharmaceutical Care at the MOH. The reports were collected from governmental hospitals and primary healthcare centers in Saudi Arabia. There were 284 hospitals and 2,380 Primary Health Care facilities.

### 2.3 Ethics approval

The study was approved by the Institutional Review Board (IRB) of the MOH, Kingdom of Saudi Arabia, for the use of the data. The national registration number with the NCBE–KACST, KSA, is H-01-R-009, dated 31/12/2023. IRB Log No:23-71 E.

### 2.4 Data source

An electronic medication error reporting system managed by the General Department of Pharmaceutical Care at the MOH was progressively enhanced during the study period. Between 2020 and 2023, the system underwent multiple upgrades to improve security, reporting efficiency, and national integration in collaboration with the Saudi Patient Safety Center. The list of biologic and biosimilar medications examined in this study was sourced from the official SFDA database, specifically the version dated August 6, 2023, comprising a total of 239 medications. Duplicate entries were removed, and all relevant biologic and biosimilar medications were included for review with the reported errors.

### 2.5 Variables

Each report documented key characteristics of the medication error itself, including the year it occurred, the stage within the medication use process (such as prescribing, dispensing, or administration) (Table 1) and the severity of the patient outcome according to the National Coordinating Council for Medication Error Reporting and Prevention (NCC MERP) harm classification scale (16). To enhance accountability and facilitate learning, the reports also documented the role of the healthcare professional responsible for detecting or reporting the error (pharmacists, physicians, nurses, or others), as well as the contributing factors that may have led to the event. Patient demographics, such as age and gender, were recorded for every reported incident.

**Table 1:** Definition of MEs with the stage (17).

Type of MEs	Definition
Prescribing errors	Errors that occurred in the prescribing decision or prescription-writing process.
Transcribing error	Deviations in transcribing medication orders from the previous prescription step.
Dispensing errors	An inconsistency between the drug prescribed and the drug dispensed to a patient.
Administration errors	On errors, Deviation from the prescriber’s medication order as written on the patient’s chart.
Monitoring errors	An error occurs when a prescribed medicine is not monitored in a way that would be considered acceptable in routine general practice.

### 2.6 Data management

Each medication error report was designed to capture structured and detailed information to support medication safety monitoring by the MOH. Although there were slight variations in the reporting systems across different years, the structure outlined above consistently represented the primary set of data fields collected throughout the study period.

To ensure data consistency for cross-year analysis:

- The contributory factors were reclassified and standardized for all years.
- Patient age was redefined into two categories: under 18 years and 18 years and above, due to inconsistencies in age groupings within the source data and the absence of a uniform reference.
- Staff roles were consolidated, with pharmacy technicians included in the pharmacist category.
- Additionally, roles such as quality management coordinator, medication safety officer, and total quality management staff were grouped under pharmacists to accurately reflect their functional responsibilities in the oversight of medication safety.
- Prior to analysis, the dataset underwent systematic cleaning procedures. Duplicate records were identified using unique report identifiers and removed. Missing or incomplete variables were retained and explicitly categorized as “Not recorded” to preserve transparency. Data consistency checks were performed across years to ensure uniform variable definitions following reclassification. Random samples of records were manually cross-verified to confirm the accuracy of variable mapping and recoding. To further enhance methodological rigor, these procedures were guided by the consolidated digital health Data Quality–Dimension and Outcome (DQ-DO) framework (18), which defines data quality across six dimensions: accessibility, accuracy, completeness, consistency, contextual validity, and currency. Accessibility was supported through structured electronic extraction from the national reporting system. Accuracy was strengthened through duplicate removal, structured recoding, and manual verification to ensure that data reflected the original reported events. Completeness was maintained by retaining missing variables and transparently reporting them as “Not recorded.” Consistency was ensured through cross-year harmonization of contributory factors, age categorization, and professional role classification. Contextual validity was preserved by aligning variable restructuring with the study objectives to ensure fitness for surveillance-based analysis. Currency was addressed by clearly defining the study period (2020–2023), presenting annual reporting trends, and acknowledging system enhancements across study years to maintain temporal integrity.

### 2.7 Data analysis

All analyses were descriptive and performed using Microsoft Excel. Given the surveillance-based design of this study and the absence of reliable medication utilization data (e.g., prescribing volume or patient exposure denominators), calculation of incidence rates or inferential comparisons between biologic and biosimilar medications was not methodologically appropriate. Therefore, results are presented as frequencies and percentages to describe reporting patterns rather than to estimate comparative risk.

## 3 Results

**Figure 1:**
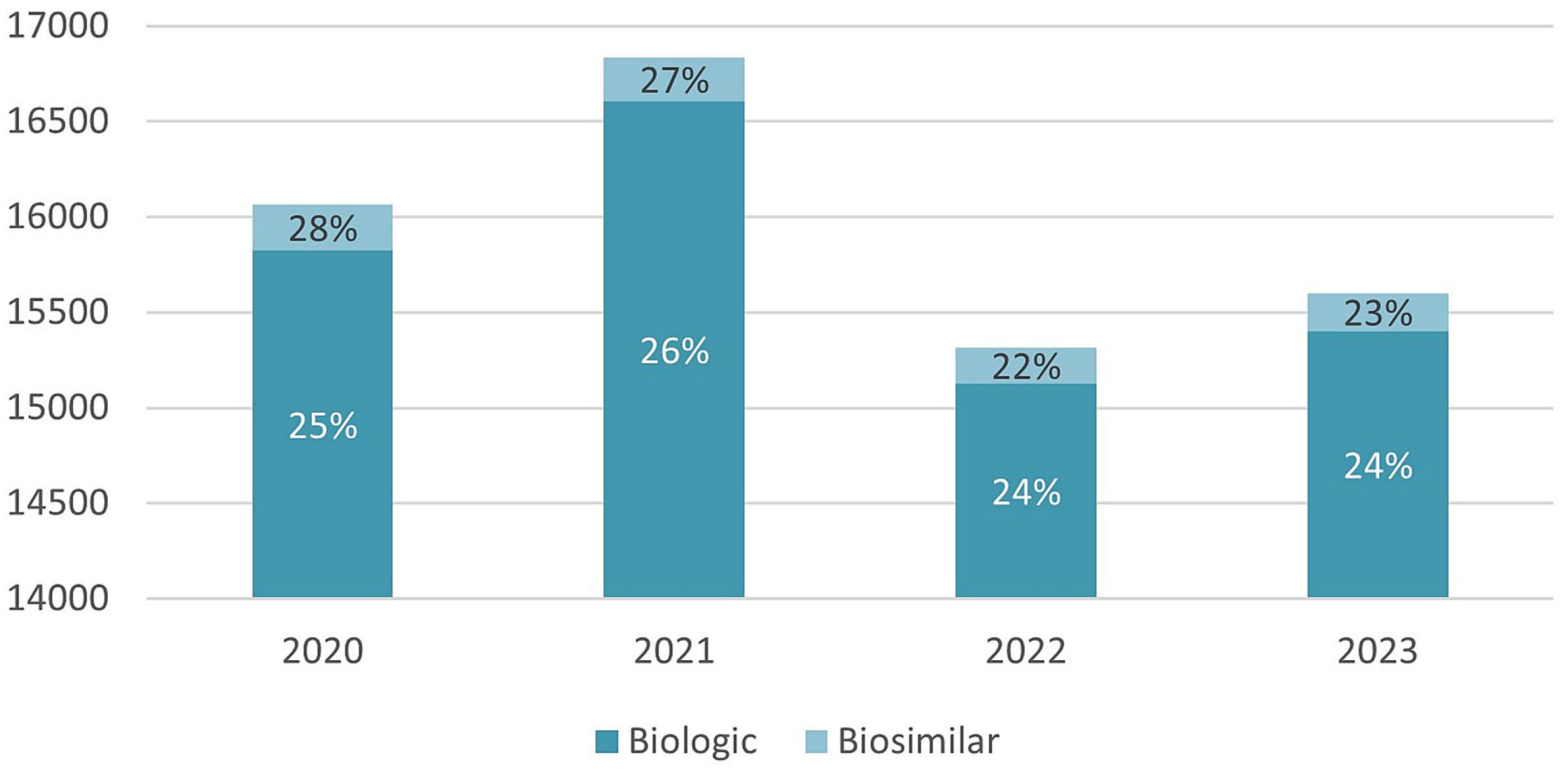
The annual incidence of reported medication errors related to biologic and biosimilar medications from 2020 to 2023.

A total of 63,817 medication error reports were identified during the study period. Of these, 62,971 (99%) were related to biologic medications and 846 (1%) to biosimilars. Denominators vary across tables due to missing or unrecorded data in certain variables and the possibility of selecting multiple response categories (e.g., stage of error and contributory factors). All variables were analyzed and presented separately for biologic and biosimilar medications. Accordingly, percentages were calculated using the total number of available reports within each respective medication category (biologic: n = 62,971; biosimilar: n = 846) and for each specific variable, unless otherwise specified. As shown in Figure 1, a variable annual trend in reported medication errors was observed between 2020 and 2023. A peak occurred in 2021, accounting for 26% of biologic-related reports and 27% of biosimilar-related reports during the study period. This was followed by a decline in 2022 and a slight increase in 2023. The figure presents the annual number of reported errors, while percentages reflect the proportion of total reports within each medication category across the four years—stages of medication errors associated with biologics and biosimilars.

Most medication errors associated with both biologic and biosimilar medications occurred during the prescribing stage, with 51,685 reports for biologics and 740 for biosimilars, representing the largest proportion of all error stages. The second most prevalent stage for biological errors was transcribing (6, 157 reports), followed by administration (3,302 reports) and dispensing (3,029 reports). A similar pattern was observed for biosimilars, although with significantly fewer reports in each category. Notably, errors during the monitoring stage and those categorized as “other” (including preparation, counseling, and storage-related issues) were relatively infrequent, although still present. From 2020 to 2022, the reporting system permitted the selection of multiple error stages within a single report. This approach likely captured complex or multi-step errors (e.g., prescribing and administration combined), and consequently, the sum of stage-specific counts may have exceeded the total number of distinct error reports for those years (Table 2).

**Table 2:** Distribution of medication errors by stages for biologic and biosimilar medications.^*^

Which stage did the error occur	Biologic	Biosimilar
Prescribing	51,685 (76%)	740 (79%)
Transcribing	6,157 (9%)	45 (5%)
Dispensing	3,029 (4%)	36 (4%)
Administration	3,302 (5%)	28 (3%)
Monitoring	999 (1%)	14 (1%)
Other (Preparation, Counseling, Hazardous Situations Only, storage)	1962 (3%)	63 (7%)
Not recorded	702 (1%)	13 (1%)
Total	67,836 (100%)	939 (100%)

^*^From 2020 to 2022, the reporting system permitted the selection of multiple error stages for each report. Consequently, the total counts may surpass the number of distinct error cases.

### 3.1 Severity of medication errors associated with biologics and biosimilars

On Table 3, Category B was the most prevalent in both classes, encompassing 43,788 biologic-related errors and 586 biosimilar-related errors, respectively. This was followed by Category A, which accounted for 17,215 and 247 errors.

**Table 3:** Distribution of medication errors by outcome severity (Categories A–I) for biologic and biosimilar medications.

What was the error outcome category?	Biologic	Biosimilar
Category A: Circumstances or events that have the capacity to cause error	17,215 (27.34%)	247 (29.20%)
Category B: An error occurred, but the error did not reach the patient (An “error of omission” does reach the patient)	43,788 (69.54%)	586 (69.27%)
Category C: An error occurred that reached the patient, but did not cause the patient harm	1724 (2.74%)	13 (1.54%)
Category D: An error occurred that reached the patient and required monitoring to confirm that it resulted in no harm to the patient and/or required intervention to preclude harm	173 (0.27%)	-
Category E: An error occurred that may have contributed to or resulted in temporary harm to the patient and required intervention	36 (0.06%)	-
Category F: An error occurred that may have contributed to or resulted in temporary harm to the patient and required initial or prolonged hospitalization	14 (0.02%)	-
Category G: An error occurred that may have contributed to or resulted in permanent patient harm	2 (0.00%)	-
Category I: An error occurred that may have contributed to or resulted in the patient’s death	3 (0.00%)	-
Not recorded	16 (0.03%)	-

### 3.2 Distribution of reported medication errors by reporter role

**Figure2:**
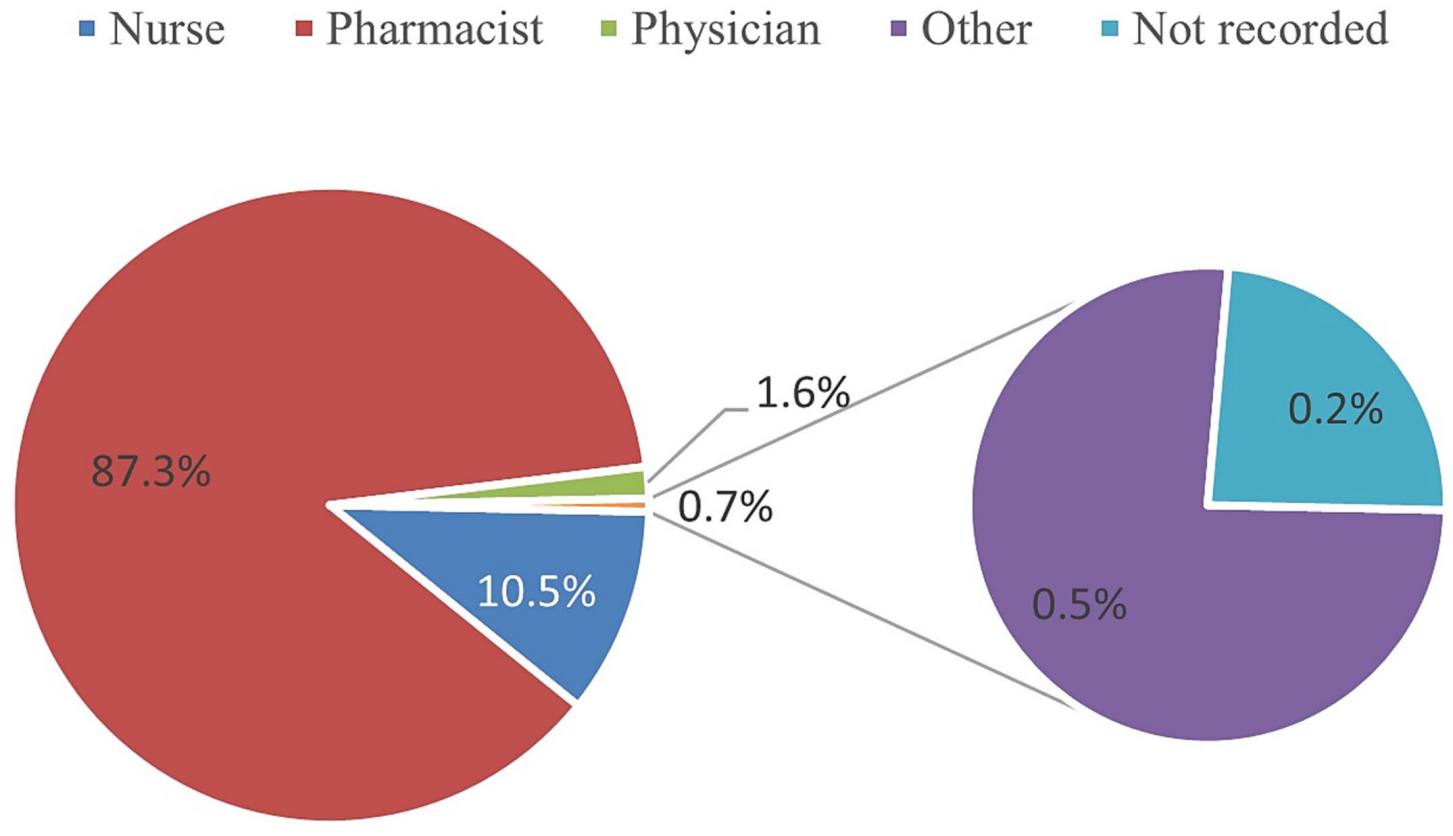
Distribution of reported medication errors by reporter role (all drug classes).

Pharmacists accounted for the majority of medication error reports, comprising 87% of all submissions. Nurses contributed to 10% of the reports, whereas physicians accounted for 2%. Reports from other healthcare personnel and unrecorded entries each constituted less than 1% (Figure 2).

### 3.3 Distribution of reported medication errors by patient age and gender

#### 3.3.1 Age category

In the context of medication errors, a significant proportion is associated with patients aged > 18 years. Specifically, 67.83% of errors related to biologic products and 74.53% of those related to biosimilar products involved adult patients (Table 4).

**Table 4:** Distribution of medication error reports by patient age category for biologic and biosimilar medications.

Age category	Biologic (N)	%	Biosimilar (N)	%
> 18 years	42,663	67.83%	600	74.53%
< 18 years	4,336	6.89%	47	5.84%
Not recorded	15,896	25.27%	158	19.63%
Total	62,895	100.00%	805	100.00%

#### 3.3.2 Gender category

Table 5 presents the gender distribution of reported medication errors for biologic medications, which exhibited a relatively balanced pattern, with 47.98% of reports involving female patients and 47.76% involving male patients, while 4.26% of reports had not recorded gender. This near-equal distribution may reflect the widespread clinical use of biologics in all genders. In contrast, errors related to biosimilars were more frequently associated with female patients (58.27%) than with male patients (41.73%).

**Table 5:** Distribution of medication error reports by patient gender for biologic and biosimilar medications.

Gender	Biologic	%	Biosimilar	%
Female	28,243	47.98%	493	58.27%
Male	28,116	47.76%	353	41.73%
Not recorded	2,509	4.26%	0	0.00%
Total	58,868	100.00%	846	100.00%

### 3.4 Factors associated with medication errors for biologic and biosimilar medications

For biologic medications, the most frequently reported contributory factors were (Table 6):

- Insufficient staff knowledge, experience, or education (22%)
- Staffing and workflow-related challenges, such as workforce shortages or high workload (18%)
- Process-related issues across the medication-use continuum, including prescribing, dispensing, administration, and monitoring (12%)

**Table 6:** Contributory factors associated with medication errors for biologic and biosimilar medications.*

Factors	Biologic	Biosimilar
Lack of Staff Knowledge, Experience, Education	13,588 (22%)	126 (15%)
Staffing or Workflow-Related Factors (Shortage of Staff, High Workload…)	11,363 (18%)	79 (9%)
Attitude-related factors (from staff, patient, caregiver…)	2,981 (5%)	43 (5%)
Lack of policies, procedures, and/or protocols	5,057 (8%)	216 (26%)
Failure to adhere to work policy, procedure, and guidelines	1,452 (2%)	10 (1%)
Environmental factors (lighting, noise, interruption, small or crowded working area)	2,776 (4%)	16 (2%)
Prohibited Abbreviation	3,338 (5%)	14 (2%)
Problem related to the electronic system	4,307 (7%)	37 (4%)
Poor Handwriting	2,375 (4%)	37 (4%)
Patient information/record unavailable/inaccurate	3,527 (6%)	24 (3%)
Factors associated with the medication use process (Prescription, dispensing, administration, and monitoring)	7,460 (12%)	182 (22%)
Other	3,627 (6%)	53 (6%)
Total	61,851 (100%)	837 (100%)

*From 2020 to 2022, the reporting system permitted the selection of contributory factors for each report. Consequently, the total counts may surpass the number of distinct error cases.

In contrast, biosimilar-related errors were more commonly associated with the following (Table 6):

- Lack of defined policies, procedures, or protocols (26%)
- Medication-use process errors (22%)
- Knowledge or experience gaps among staff (15%)

It is essential to recognize that multiple contributory factors may be identified in each case report. Consequently, the aggregate frequency of reported factors surpassed the number of distinct medication error cases.

The top six medications associated with reported medication errors were as follows

Table 7 provides a comprehensive comparison of the six leading medications associated with medication errors, encompassing four biologic agents (Enoxaparin, Heparin, Insulin Aspart, and Insulin Glargine) and two biosimilars (Adalimumab and Teriparatide). The prescribing stage was identified as the most prevalent error stage for all medications. All medications predominantly fell under Category B, indicating that the errors did not reach the patient. Notably, Heparin (82%) and enoxaparin (72%) exhibited the highest proportions in this category, suggesting the presence of robust interception systems. The adult population (>18 years) was the most frequently affected age group for all medications. Pharmacists were the primary reporters for all medications, accounting for > 85% of the reports in each case. A gender-specific trend was noted, with female patients more often involved in errors related to insulin and teriparatide, while male patients were more commonly linked to cases involving heparin. The contributory factors varied by medication. Knowledge gaps were most frequent for anticoagulants and insulin, while policy/procedure lack were more prominent for biosimilar medications.

**Table 7:** Summary of the top six medications associated with reported medication errors, including four biologic medications (Enoxaparin, Heparin, Insulin Aspart, Insulin Glargine) and two biosimilar-related medications (Adalimumab, Teriparatide).

Variables	Biologic				Biosimilar	
	Enoxaparin, *N* (%)	Heparin, *N* (%)	Insulin Glargine, *N* (%)	Insulin Aspart, *N* (%)	Adalimumab, *N* (%)	Teriparatide, *N* (%)
Number of medication errors	34,155 (54%)	6,483 (10%)	4,526 (7%)	4,255 (7%)	481 (57%)	232 (27%)
Stage	Prescribing 28,080 (74%)	Prescribing 5,110 (67%)	Prescribing 3,810 (84%)	Prescribing 3,474 (72%)	Prescribing 416 (76%)	Prescribing 202 (84%)
Outcome category	Category B 24423 (72%)	Category B 5311 (82%)	Category B 2939 (65%)	Category B 2888 (68%)	Category B 353 (73%)	Category B 163 (70%)
Healthcare	Pharmacist 29,206 (86%)	Pharmacist 5,790 (89%)	Pharmacist 4,030 (89%)	Pharmacist 3,897 (92%)	Pharmacist 466 (97%)	Pharmacist 226 (97%)
Age category	> 18 years 24,470 (72%)	> 18 years 4,284 (66%)	> 18 years 3,408 (75%)	> 18 years 2,575 (61%)	> 18 years 342 (71%)	> 18 years 168 (72%)
Gender category	Female 21,360 (63%)	Male 3,859 (60%)	Male 2,338 (52%)	Female 2,222 (52%)	Male 245 (51%)	Female 174 (75%)
Factors	Lack of Staff Knowledge, Experience, Education 6,933 (21%)	Lack of Staff Knowledge, Experience, Education 2,453 (38%)	Staffing or Workflow-Related Factors (Shortage of Staff, High Workload…) 981 (22%)	Lack of Staff Knowledge, Experience, Education 989 (24%)	Lack of policies, procedures, and/or protocols 147 (31%)	Lack of policies, procedures, and/or protocols 53 (23%)

*Percentage calculated: From 2020 to 2022, the reporting system permitted the selection of multiple error stages and contributory factors for each report. Consequently, the total counts may surpass the number of distinct error cases.

## 4 Discussion

### 4.1 Overview and interpretation of key findings

Limited published data have examined medication errors specifically associated with biologic and biosimilar medications at a nationwide level within a unified reporting system. The present study provides a comprehensive real-world national analysis of medication errors reported to the Saudi MOH over a four-year period. A total of 63,817 medication error reports were documented during the study period, of which 62,971 (99%) were related to biologics and 846 (1%) to biosimilars. Our findings indicate that the prescribing stage constituted the predominant phase of errors, representing 76% of biologic related and 79% of biosimilar related medication errors. This observation aligns with existing national and international evidence. A nationwide study in Saudi Arabia found that 84.8% of medication errors occurred during the prescribing phase, indicating the considerable dependability of this stage in the medication-use process (5). A systematic review in the United Kingdom regarding hospitalized children revealed a significant prevalence of prescribing errors (19). Furthermore, international reviews have highlighted that the prescribing phase represents an essential vulnerability in healthcare systems. To mitigate these errors, several strategies have been proposed, such as electronic prescribing, computerized decision support systems, and the active participation of pharmacists (20). These findings highlight the necessity of prioritizing interventions during the prescribing stage to improve medication safety. Furthermore, international reviews have highlighted that the prescribing phase represents an essential vulnerability in healthcare systems. To mitigate these errors, several strategies have been proposed, such as electronic prescribing, computerized decision support systems, and the active participation of pharmacists (16). These findings highlight the necessity of prioritizing interventions during the prescribing stage to improve medication safety.

Furthermore, Category B errors, characterized as errors that occurred but did not affect the patient, predominated our findings. While the predominance of Category B errors may partly reflect the heightened monitoring and interception mechanisms applied to high-alert medications, such as some of the biologics (21). It is also consistent with well-documented patterns observed in voluntary medication error reporting systems. Evidence indicates that incident reporting databases are frequently dominated by near-miss and no-harm events, as these are more readily detected and reported within structured safety systems rather than representing the true distribution of harmful events (22, 23). These findings suggest that a high proportion of Category B errors may reflect detection practices and safety surveillance maturity rather than a true predominance of clinically insignificant errors. The WHO has further emphasized that data derived from reporting and learning systems should not be interpreted as measures of true incidence rates, as they are shaped by system design, accessibility, and reporting behavior (23). Therefore, the high proportion of Category B events observed in this study should be understood within the broader context of reporting culture and system-level detection practices, rather than being interpreted as evidence of superior safety performance. Notably, pharmacists were the primary reporters of medication errors in our study, which is consistent with both national and international evidence. In a nationwide Saudi study, 75.9% of errors were detected by pharmacists (5). Similarly, the United Kingdom and in the United States have demonstrated the central role of pharmacists in error detection and prevention (24, 25). However, this predominance should be interpreted cautiously. Voluntary reporting systems inherently capture only a proportion of actual medication errors, and the types of events reported are influenced by professional engagement, perceived responsibility, and institutional reporting culture (26, 27). Qualitative evidence further indicates that attitudes toward incident reporting vary significantly across professional groups, with factors such as fear of blame, time constraints, and perceptions of role responsibility influencing reporting behavior (28). Such variations may contribute to the disproportionate representation of certain healthcare professionals within medication error databases, reflecting differences in reporting practices rather than true differences in error occurrence.

Our study found that the majority of reported medication errors occurred in adults (>18 years), comprising 67.8% of biologic-related errors and 74.5% of biosimilar-related errors. This pattern aligns with international evidence, as adult populations, especially those on multiple or high-risk medications, are disproportionately impacted by medication errors, highlighting the necessity for improved safety strategies for this particular population (29). Insulin and anticoagulants, specifically enoxaparin and heparin, were identified as the most common causes of reported medication errors in our study. This finding aligns with the classification by the Institute for Safe Medication Practices (ISMP), which identifies insulin and anticoagulants as high-alert medications due to their narrow therapeutic windows and the potential for significant patient harm (21). A multi-center analysis of the UK National Reporting and Learning System indicated that insulin represented the highest percentage of high-risk medication errors, followed by anticoagulants (30). A systematic review of high-alert medications confirmed that insulin and anticoagulants are consistently among the most frequently implicated in harmful medication errors globally, emphasizing their importance as safety priorities (31). Three convergence of these findings highlights the necessity for targeted safety strategies, such as standardized prescribing protocols. Our study found that biosimilars were associated with fewer errors. However, specific medications such as adalimumab and teriparatide exhibited vulnerabilities primarily attributable to a lack of clear policies, procedures, and protocols governing their use. Randomized trials provide evidence that switch from biologics to biosimilars is clinically safe and maintains efficacy (32). Additionally, reviews on biosimilar-to-biosimilar switching suggest that the primary challenges arise from regulatory and operational challenges rather than pharmacological aspects (33). Our findings indicate that the errors associated with biosimilars are primarily the result of systemic deficiencies rather than inherent risks of the products. Contributing factors for errors with other medications included staff knowledge deficits, lack of experience, and challenges within the medication-use process. These determinants are consistent with the findings of a nationwide study from Saudi Arabia, which highlighted similar system and human-related limitations as major contributors to medication errors (5).

### 4.2 Future research and policy implications

Future research should prioritize prospective evaluations of interventions aimed at mitigating medication errors specific to biologic therapies, including assessments of the real-world effectiveness of biosimilar switching protocols. Additionally, it is necessary to investigate the sociocultural barriers that impede reporting among nurses and physicians. Future large studies are needed to assess the causality between these biologic and biosimilar medication errors and adverse outcomes. Given the relevance of policy clarity to biosimilar safety, further studies on biosimilar policies are warranted to provide useful guidance for achieving consistency across institutional levels for suitable harmonization.

### 4.3 Study strengths and limitations

One of the significant strengths of this research lies in its use of multiyear nationwide data obtained from the official MOH reporting systems, offering a comprehensive perspective on evolving trends in medication safety. Furthermore, the study’s focus on the analysis of biologics and biosimilar medications, whose safety profiles are not regularly examined in the context of typical hospital errors, further enhances its strengths. Although this study utilized comprehensive national data from hospitals associated with the MOH across Saudi Arabia, its implications may be limited to health systems with analogous medication safety, reporting, and regulatory frameworks. Nevertheless, the observed trends in high-alert medication errors and contributory factors, such as lack of staff knowledge and systemic inefficiencies, align with the global literature on this subject, potentially rendering them applicable to other hospital settings. This study has several important limitations that should be considered when interpreting the findings. The use of multiple electronic systems over several years may have led to inconsistent data and the voluntary nature of reporting could have led to underreporting. Importantly, reported medication errors do not represent true incidence rates but rather reflect detected and voluntarily reported events. Therefore, findings may be influenced by institutional reporting culture, variations in safety awareness, and detection practices across facilities. The differences in how errors are classified and the factors that contribute to them highlight the impact of systemic and contextual challenges within MOH institutions (34). Retrospective reclassification of age, staff roles, and contributory factors was conducted to ensure comparability. Reclassification is also subject to interpretative bias. Furthermore, the identification of biologic and biosimilar medications was based on the 2024 SFDA official registry of approved products. This updated classification was retrospectively applied to reports spanning 2020–2023 to ensure consistent categorization of originator biologics and biosimilars. However, as regulatory listings and product classifications may evolve over time, this retrospective alignment may have introduced potential misclassification bias, particularly for products whose approval or classification status changed during the study period. This methodological constraint, specifically the retrospective application of updated medication classifications, reflects a common challenge reported in the pharmacovigilance literature (35).

## 5 Conclusion

This study offers a comprehensive national evaluation of medication errors associated with biologic and biosimilar products reported to the Saudi MOH between 2020 and 2023. High-alert medications, particularly insulin and anticoagulants, consistently ranked among the top error related medications, highlighting the need for targeted interventions.

## Data Availability

The datasets analyzed in this study are not publicly available due to institutional and regulatory restrictions, as they were obtained from a national medication error reporting system and contain sensitive and confidential information. Access to the data may be considered upon reasonable request and subject to approval from the relevant authority.
